# Tomotherapy as an Alternative Irradiative Treatment for Complicated Keloids

**DOI:** 10.3390/jcm9113732

**Published:** 2020-11-20

**Authors:** Yu-Fang Lin, Pei-Wei Shueng, Tyng-Luen Roan, Dun-Hao Chang, Yen-Chen Yu, Che-Wei Chang, An-Ta Kuo, Yo-Shen Chen, Hsiu-Wen Hsiao, Hui-Ju Tien, Chen-Hsi Hsieh

**Affiliations:** 1 Division of Radiation Oncology, Department of Radiology, Far Eastern Memorial Hospital, New Taipei 22060, Taiwan; jay7798@femh.org.tw (Y.-F.L.); shueng@femh.org.tw (P.-W.S.); ep0207@femh.org.tw (H.-W.H.); catju@femh.org.tw (H.-J.T.); 2 Faculty of Medicine, School of Medicine, National Yang-Ming University, Taipei 11221, Taiwan; 3 Department of Plastic Surgery, Far Eastern Memorial Hospital, New Taipei 22060, Taiwan; troan@femh.org.tw (T.-L.R.); femh93940@femh.org.tw (D.-H.C.); femh91203@femh.org.tw (Y.-C.Y.); d08945011@ntu.edu.tw (C.-W.C.); plastic@femh.org.tw (A.-T.K.); prs001@femh.org.tw (Y.-S.C.); 4 Institute of Traditional Medicine, School of Medicine, National Yang-Ming University, Taipei 11221, Taiwan

**Keywords:** electron beam radiotherapy, gafchromic film, helical tomotherapy, keloid

## Abstract

The aim of this study was to investigate the treatment of complicated keloids with helical tomotherapy (HT) and electron beam radiotherapy. From July 2018 to September 2018, 11 patients with 23 keloid lesions treated with HT were enrolled. Additionally, 11 patients with 20 lesions treated with electron beam radiotherapy in the same period were enrolled. Patients in both groups were treated within 24 h after surgical excision of the keloid lesion with 13.5 Gy in three consecutive daily fractions. The median follow-up period was 15 months. The local control rate was 91.3% and 80% in the HT group and the electron beam group, respectively. No acute adverse effects were observed in either group, but most patients exhibited pigmentation. No radiation-induced cancer occurred in these patients up to the time of this report. Pain and pruritus improved for all patients and more obviously for three patients with complicated keloids treated with HT. The measured surface dose was 103.7–112.5% and 92.8–97.6% of the prescribed dose in the HT group and the electron beam group, respectively. HT can be considered an alternative in cases where it is not feasible to use multiple electron fields, due to encouraging clinical outcomes.

## 1. Introduction

Keloids are disfiguring fibroproliferative lesions that grow beyond the boundaries of the original wound scar and can significantly impair the quality of life of affected individuals [1]. Keloids can occur at any age but most commonly occur during the second and third decades of life [2]. Affected patients may experience itching, pain, impaired range of motion, and interference with normal daily activities, and keloids can be complicated by ulceration, bleeding, and infection [2].

Various adjuvant keloid treatment strategies have been tested, including surgical excision, pressure treatment, intralesional steroids, interferon and 5-fluorouracil injections, laser therapy, cryotherapy, and silicone gel sheeting [3]. However, the relapse rate is greater than 50% for patients receiving these treatments [3,4]. According to the international recommendations for scar management, postoperative radiotherapy is considered to be an effective method available for keloids to decrease the relapse rate to 20% [4,5,6,7,8,9]. One of the radiation-based modalities used for keloids is electron beam irradiation [10]. Nevertheless, electron beam irradiation exhibits an inhomogeneous dose distribution, especially when the treated area is irregular, has a large slope [11], or is in a junctional field [12].

Helical tomotherapy (HT, Accuray, Inc., Madison, WI, USA) is an advanced modality using a 6 MV photon beam linear accelerator. Compared with volumetric-modulated arc therapy and intensity-modulated radiotherapy (IMRT) for clinical postmastectomy radiotherapy, in HT, the surface dose is not reduced [13]. Additionally, the special design of HT to restrict exposure to only obliquely incident photon beams can decrease damage to critical organs while increasing the superficial dose [14,15].

According to the potential benefits of HT in treating superficial lesions, we retrospectively assessed the efficacy and dose verification in patients with keloids who underwent surgical excision followed by HT or electron beam irradiation.

## 2. Materials and Methods

### 2.1. Patient Selection

From July 2018 to September 2018, eleven patients with 23 keloids treated with HT and 11 patients with 20 lesions treated with electron beam radiotherapy (Elekta VERSA HD linac accelerator, Elekta Oncology Systems Ltd, Crawley, UK) were enrolled. Retrospective data were collected after receiving approval from the Institutional Review Board of the Far Eastern Memorial Hospital (FEMH-IRB-109069). The need for informed consent was waived by the IRB of FEMH due to the research involving no more than minimal risk to subjects.

### 2.2. CT Simulation for HT

**Figure 1:**
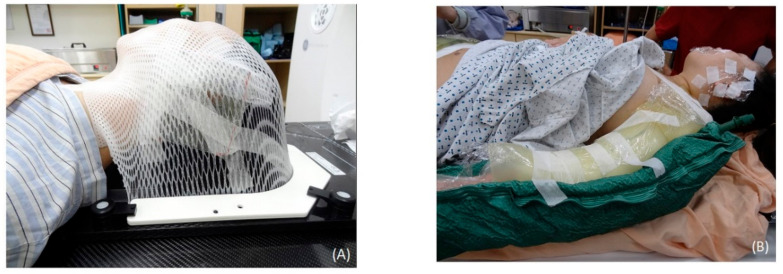
CT simulation setup conditions for patients with keloids in different areas after resection. (**A**) A patient with a keloid on the left ear was immobilized by a U-frame head and neck immobilization system (CIVCO Radiotherapy, Orange City, IA, USA), with her face turned to the contralateral side to expose the operative ear. (**B**) A patient with complicated keloids on the body was immobilized by a Body Vac Cushion (Klarity Medical, Newark, OH, USA) in the supine position.

The patients underwent CT (Discovery CT590 RT, GE Healthcare, Chicago, IL, USA) with 2.5 mm slice spacing, and then the image sets were transferred to a treatment planning system (Pinnacle3, version 9.8.1, Philips Medical Systems, Madison, WI, USA) for targeting and organ delineation. The patients who underwent head keloidectomy were immobilized by a U-Frame head and neck immobilization system (CIVCO Radiotherapy, Orange City, IA, USA) with the face turned to the contralateral side (Figure 1A). A Body Vac Cushion (Klarity Medical, Newark, OH, USA) was used to immobilize the whole body of patients with keloids on the torso or extremities (Figure 1B). Additionally, a 1 cm bolus was added to the surgical area to provide an adequate surface dose to the scar.

### 2.3. Target Contouring, Treatment Planning, and Treatment by HT

The clinical target volume (CTV) was defined as the area encompassing the surgical bed subcutaneously 0.8 cm from the skin plus a 1 cm width margin. The inner margin of the planning target volume (PTV) was 0.5 cm, and the outer margin of the PTV was defined by a three-dimensional (3D) expansion of 0.5 cm. The plan was created using the Tomotherapy Hi Art Planning system (version 5.1.3, Tomotherapy, Inc., Madison, WI, USA). For HT planning, three parameters should be selected: the field width (the slice thickness of the radiation field projected at the isocenter along the gantry rotation axis) was set to 5.048 cm, the pitch (the couch movement relative to the field width during one gantry rotation) was set to 0.2–0.3, and the modulation factor (the ratio between the maximum number of opening leaves and the average number of opening leaves in active gantry rotation) was set to 2.6–3.0. The calculation grid was 0.195 × 0.195 cm. Patients were treated by HT with 13.5 Gy in 3 consecutive daily fractions within 24 h after surgical excision of the keloid lesion.

### 2.4. Plan Evaluation for HT

#### 2.4.1. Paddick Conformity Index (PCI) and Uniformity Index (UI)

The PCI was originally proposed by Paddick [16] to evaluate the tightness of fit of the PTV to the prescription isodose volume in treatment plans and was calculated as follows:

PCI = (TV_PIV_^2^)/(TV × PIV)

where TV is the PTV volume, PIV is the treated volume enclosed by the prescription isodose surface, and TV_PIV_ is the volume of the PTV within the prescribed isodose. A PCI closer to unity means greater conformity of the dose distribution to the target volume.

The UI was defined as D5%/D95%, where D5% and D95% is the minimum dose delivered to 5% and 95% of the PTV, respectively, as previously reported [17].

#### 2.4.2. Dose Sparing for Organs at Risk (OARs) for HT

The mean and maximum doses were reduced to the lowest possible for all critical organs. For keloids in the head area, the critical organs included the lens, eyeball, optic nerve, optic chiasm, inner ears, parotids, and brain. For keloids on the chest and abdomen, the critical organs included the trachea, esophagus, whole lungs, heart, intestine, liver, stomach, and kidneys. The bladder, rectum, uterus, and femur bones were critical organs for keloids in the pelvis area. The cervical spine, thoracic spine, lumbar spine, and iliac, pelvic, and femur bones were contoured and expected to receive a mean dose less than 4.0 Gy to avoid bone marrow toxicity [18].

#### 2.4.3. Dose Prescription Policy

To ensure dose coverage of target volume, the primary aim in HT plan was to achieve 100% prescription dose with 95% PTV volume (D_95_ of PTV = 13.5 Gy). Besides this, the maximum dose of PTV should be lower than the 115% of prescription dose.

### 2.5. Treatment with Electron Beam Irradiation

The surgical incision area with a 1.5 cm margin was treated by electron beam irradiation (6 MeV) with custom Cerrobend blocks. The prescribed dose was 13.5 Gy in 3 consecutive daily fractions with a 90% isodose line and a 3 mm bolus. All lesions underwent surgical excision followed by electron beam irradiation within 24 h.

### 2.6. Surface Dose Measurement

Gafchromic EBT3 films (Ashland ISP, Wayne, NJ, USA) in the form of 2.5 × 3.0 cm^2^ sheets were used to measure the surface dose of the keloid lesions. The EBT3 films were digitized by using an Epson 11000XL scanner (Seiko Epson Corporation, Nagano, Japan) and analyzed with Film QA Pro software (Film QA Pro 2015, version 5.0, Ashland, Inc., Wayne, NJ, USA) [19]. In all investigations, EBT3 films were scanned within 24 h after irradiation. All scans were acquired in a 48-bit RGB color channel (16 bits per color) transmission mode with a resolution of 72 dpi and all available color correction options turned off. After scanning, the images were saved in the tagged image file format (tiff).

### 2.7. Outcome Assessment

In the current study, keloid patients were followed and evaluated by every 3 months. The scar was examined by the radiation oncologist and the plastic surgeon during the follow-up period. There were no post-operative treatments after radiation therapy. However, the patients received steroid injections when the scar was recurrent. Recurrence was defined as elevation of the scar above the plane of the skin or scar dehiscence and the reappearance of itching and erythema. Adverse effects included acute and chronic radiotherapy-related complications. Acute adverse effects included skin ulceration and lack of wound healing after the operation. Chronic adverse effects included skin hyperpigmentation and telangiectasia with depigmentation (occurring within a year after treatment). Considering the limited sample number in this study, the differences between the techniques were analyzed with the Wilcoxon signed-rank test (a nonparametric test) for paired samples. A *p*-value below 0.05 was considered significant.

### 2.8. Surgical Process

Most of the surgeries were done under local anesthesia, and if the lesion was large or needed further reconstruction procedure, general anesthesia was administered. Excision was performed at the margin between the lesion and the normal skin. If the scar contractures were present, the adhesion and contracture were released to demonstrate actual defect size. The reconstruction methods were selected according to the defect size and skin tension. Most of the wounds were closed primarily with long-lasting absorbable sutures (e.g., Monocryl, Poly-p-dioxanone) subcutaneously and the skin was closed with 5-0 nylon, which was removed 7–10 days later. If the defect size or skin tension were too large, local flap or skin graft was required.

## 3. Results

There were five males and six females in the HT group and four males and seven females in the electron beam group. The average age was 43 years (range, 20–75 years) in the HT group and 38 years (range, 22–66 years) in the electron beam group. The characteristics of the patients and sites are summarized in Table 1. The median follow-up time was 13 months (range, 12–14 months) in the HT group and 15 months (range, 13–16 months) in the electron beam group. In the HT group, two patients had five keloids, one patient had three keloids, two patients had two keloids, and six patients had one keloid. Additionally, there were three patients with severe keloids. There were 11 lesions in high-tension areas and 12 lesions in low-tension areas [20]. In the electron beam group, one patient had three keloids, seven patients had two keloids, and three patients had one keloid. There were 12 lesions in high-tension areas and eight lesions in low-tension areas.

**Figure 2:**
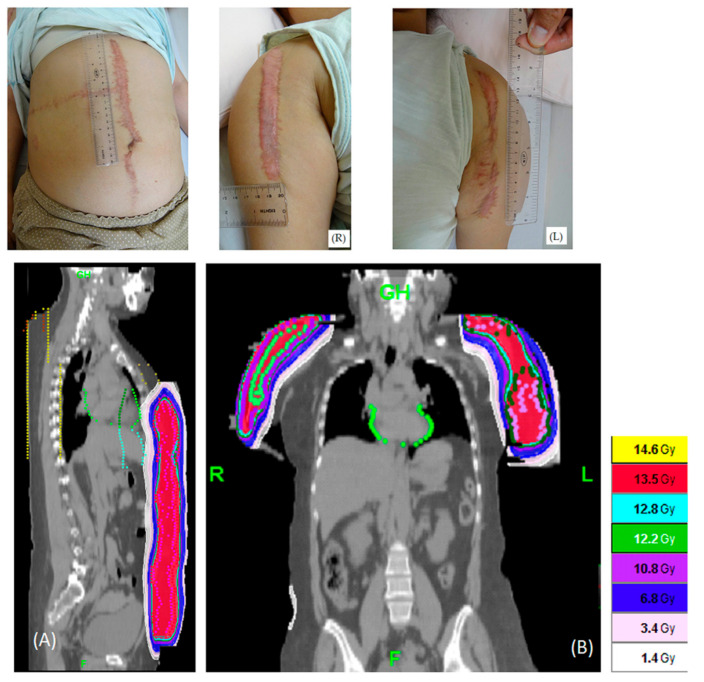
One patient had an extensive keloid caused by a car accident (up three pictures in the figure). She was treated with helical tomography within 24 h after keloid excision with 13.5 Gy in 3 fractions. (**A**) Sagittal view. (**B**) Coronal view.

**Figure 3:**
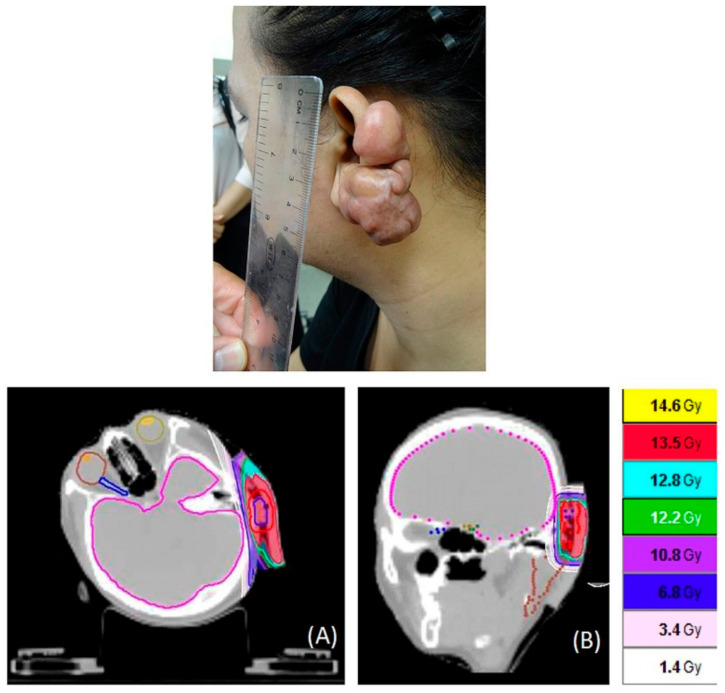
One patient had a keloid on the left ear (up picture in the figure). He was treated with helical tomography within 24 h after keloid excision with 13.5 Gy in 3 fractions. (**A**) Transverse view. (**B**) Sagittal view.

One patient in the HT group had the largest PTV, at 1159.8 mL, on the abdomen. The isodose distributions for the complex areas (torso and bilateral arms) of the patient are shown in Figure 2. The isodose distributions for the left ear are shown in Figure 3. The PCI, UI, and various OARs are listed in Table 2. The mean and maximum doses of critical organs were all under 1.8 Gy, and most were under 1 Gy in treatment planning.

The surface dose of lesions in the HT group and the electron beam group was 103.7–112.5% of the prescribed dose (range, 466.5–506.1 cGy) and 92.8–97.4% of the prescribed dose (range, 417.6–438.3 cGy), respectively. The mean dose was 486 and 427.5 Gy, respectively (Table 3). The shortest beam on time was 254.1 s for single keloid lesion, and the longest beam on time was 1271.3 s for complicated keloid, and the mean beam on time was 569.5 s for all HT patients.

**Figure 4:**
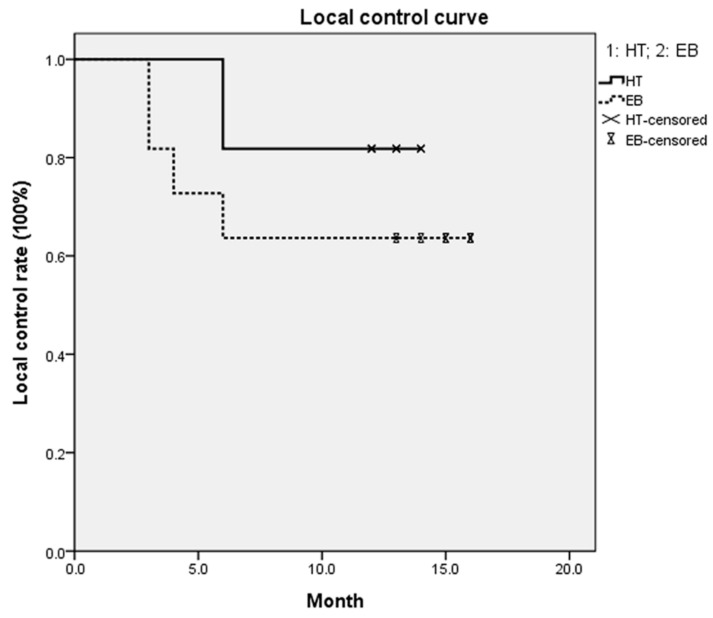
One-year local control rates for patients treated with helical tomotherapy or electron beam irradiation. There were no significant differences between the HT and electron beam (EB) groups (*p* = 0.238).

**Figure 5:**
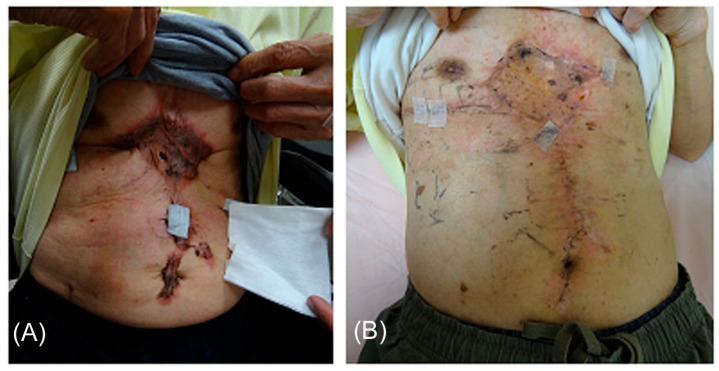
(**A**) Pre-treatment and (**B**) post-treatment images of the keloid of one patient treated with helical tomography. Post-treatment, the scar was white and not elevated. Additionally, the patient reported no tightness, itching, or painful sensations.

In the HT group, one abdominal lesion and one left arm lesion relapsed six months after irradiation. Four lesions relapsed three to six months after electron beam irradiation; one was on the scapula, one was on the left arm, and two were on the chest. The local control rate was 91.3% in the HT group and 80% in the electron beam group. The one-year local control rate was 81.8% and 63.4% in the HT group and the electron beam group, respectively (Figure 4). The *p*-value between the two groups was 0.238. There were no acute or chronic radiotherapy-related adverse effects, such as skin ulceration, lack of wound healing, or infection, in these patients (Figure 5). Skin hyperpigmentation was noted in the irradiation field for all patients, but the skin color returned to normal within a year after treatment. One patient with an ear keloid developed temporary, slight hair loss around the treatment site. No patients suffered from treatment-related cancer up to the time of this report.

## 4. Discussion

Keloids commonly adopt distinct site-specific shapes, namely the typical butterfly, crab claw, and dumbbell shapes on the shoulder, anterior chest, and upper arm, respectively [21]. These areas are highly mobile and high-tension areas [20] and are reported as the most common sites of recurrence [22]. At present, there are many therapeutic options available, including surgery, radiation, corticosteroids, 5-fluorouracil, cryotherapy, laser therapy, and cosmetic therapies [23]. The main problem of surgery for pathological scars is recurrence, and the recurrence rate is approximately 45–100% [3].

The cutaneous wound healing process includes coagulation, inflammation, proliferation, and remodeling [24]. Keloids are fibroproliferative lesions that result from abnormal wound healing [25,26]. Compared to surgery alone, radiotherapy following surgery decreases the relapse rate of keloids to 10–20% [4,5,6,7,8,9]. The possible mechanisms of keloid inhibition by irradiation include the suppression of angiogenesis, which decreases the delivery of inflammatory cytokines, and the successive inhibition of fibroblast activity, resulting in decreased collagen synthesis and thus effectively suppressing keloid development [26,27]. Additionally, irradiation potentially reduces keloid fibroblast autophagy and migration by targeting phosphatase and tensin homolog (PTEN) [28]. Moreover, irradiation not only silences many genes in keloid fibroblasts that play roles in enhancing cell proliferation and extracellular matrix production but also upregulates genes involved in promoting apoptosis and extracellular matrix degradation [29].

Since the early 1900s, radiotherapy has been used for treating keloids and has been deemed safe and effective in numerous studies [5]. One of the radiation-based modalities used for treating keloids is electron beam irradiation [10]. However, the inhomogeneity of the electron beam irradiation dose distribution in areas with a large slope does not allow the treatment of concave or convex volumes with a homogeneous dose [11,30] or a large field with a junctional position [12,31]. A dosimetric evaluation in electron beam irradiation of the total scalp showed that the dose variation in junctional regions ranged from −50% to +70% [11]. A 20–50% hot spot caused by electron scattering and a 10% cold spot caused by a shorter distance from the surface to the electron source at the junction line between photon and electron beams have been reported [12,31]. Moreover, inhomogeneity of approximately 15% can occur across the skin surface [32]. Deviations of 32–124% from the prescription dose in specific areas of the body have been reported in total skin electron beam therapy [30]. Additionally, the maximum irradiation size for electron beam is only 25 cm in the Elekta linac accelerator [33], which limited of the field of treatment. As mentioned above, HT could be considered for complicated situations such as larger treatment size (>25 cm), irregular shape, and large slope position. In the current study, HT provided an 11–15% higher surface dose to the keloid lesions than did electron beam irradiation. Additionally, the one-year local control rate was approximately 20% higher in the HT group than in the electron beam group, although the difference between the groups was not significant (*p* = 0.238). The possible reason may result from the small number of cases in the current study.

By using central core complete block to restrict exposure to only obliquely incident photon beams, thereby increasing the superficial dose and reducing the internal organ dose, in the treatment of irregularly shaped cutaneous areas where it is difficult to use multiple electron fields, HT provides impressive uniformity, conformity, and dose distribution with limited damage to critical organs [14,34,35]. In the current study, the mean and maximum doses to critical organs were under 1.8 Gy in patients treated by the HT technique. Additionally, the PCI was apparently lower than 1. In fact, the electron beam falls off rapidly after Dmax, but the photon beam falls off smoothly. It makes electron beams obliquely incident onto the surface with bolus, which results in a decreased surface dose relative to an unbolused surface, but the photon beam does not [14]. The surface dose delivered by HT for the total skin reportedly ranges from 85% to 150% of the prescribed dose [14,34,35]. In the current study, the surface dose delivered to lesions measured by EBT3 film exposure ranged from 103.7% to 112.5% of the prescribed dose in HT. Moreover, the surface dose delivered to the lips and pelvis was larger than 10% of the prescribed dose, which may have been caused by the tangential beam angles utilized in the treatment plan to spare normal tissues. In electron beam irradiation, the measured surface dose was lower than the prescribed dose in all cases, and the largest variation was observed for the ear, which may have been due to the irregular shape. Most areas showed approximately 5% error due to the small and narrow field. Underdose of the lesion could be one of the reasons for the higher recurrence rate after electron beam irradiation than after HT.

Kal et al. [36] suggested that the recurrence rate after the delivery of a biologically effective dose (BED) greater than 30 Gy and of approximately 20 Gy is less than 10% and approximately 20–25%, respectively. The BED can be calculated as follows: BED = nd [1 + d/(α/β)] [37,38], where “n” is the number of fractions, “d” is the dose per fraction, and “α” and “β” are parameters that determine the initial slope and curvature of the underlying cell survival curve. For keloids, as acute responding tissues, α/β is 10 Gy [36]. The BED for the current dose schedule (13.5 Gy in 3 fractions) was 19.57 Gy, and the recurrence rate was 8.7% for HT and 20% for electron beam radiotherapy. Additionally, higher recurrence rates are found for keloids in the chest wall and scapular and suprapubic regions. In contrast, regions with low tension, such as the earlobe, have lower recurrence rates [8,39]. However, HT provides dose homogeneity with precise depth penetration to offset the disadvantages of electron beam irradiation, and the surface dose was 103.7–112.5% of the prescribed dose. In contrast, the surface dose in similar areas treated by electron beam irradiation and measured by EBT3 films was 92.8–97.4% in our clinical experience. The underdose by electron beam irradiation indicates that the relapse rate may be higher in these patients. The recurrence rate for electron beam radiotherapy in the current study is similar to that in other studies [39,40]. As mentioned above, the treatment of keloids with HT reduces the recurrence rate as much as possible.

A frequently posed question in the treatment of benign diseases by irradiation concerns the risk of radiation-induced malignancy. De Lorenzi et al. [40] reported no cases of radiation-induced cancer after brachytherapy for keloids. Additionally, in a study that included more than 6500 patients who were treated with external radiotherapy, only five cases of potential and doubtful radiation-induced cancer were noted [41]. Additionally, the risk of inducing the development of a fatal tumor with a single dose of 13 Gy (BED = 30 Gy) is approximately 1.3 × 10^−6^ [36], which is similar to the risk of inducing the development of a fatal tumor by a computed tomography scan of the chest (4.8 × 10^−6^) [42]. Considering that the BED was approximately 20 Gy in the current study, which is lower than 30 Gy, the risk of a radiation-induced tumor seems to be limited or even negligible. Additionally, psychological factors (satisfaction with appearance, shame and suffering from the disease) and physical problems (pruritus, pain, and restricted movement) impair the quality of life of patients with keloids [43]. For those with lesions that are irregular in shape or not suitable for junctional electron beam irradiation, HT provides an opportunity for the treatment of complex keloids, which merits further exploration.

There are some limitations to the present study. First, the sample size of patients was limited, making statistical conclusions very tentative. Therefore, enrolling a larger sample of patients to confirm the effects of HT for complicated keloid treatment is warranted in the future. Second, quality-of-life assessments were not included in the current study due to the limited number of patients. Third, Maemoto H et al. [44] reported that the local control rates of same keloid lesions could be 93% at 1 year and 68% at the 10th year. However, the median follow-up time in the current study was 13 and 15 months in the HT and electron beam group, respectively. In the future, a longer following time to confirm the current observation is warranted.

## 5. Conclusions

The current study is the first to perform dose verification on keloid scars treated with electron beam irradiation and HT. HT provided a higher surface dose with precise conformity and depth penetration and a higher local control rate than electron beam irradiation for patients with complex or difficult-to-treat keloids. Considering the published data, the clinical results, and the limited and reversible toxicity of HT, it can be considered an alternative for treating large, convex, cutaneous areas that are difficult or not feasible for any reason to treat using multiple electron fields.

## Figures and Tables

**Table 1:** Patient characteristics.

	HT Group (Lesion Number, *n* = 23)	Electron Beam Group (Lesion Number, *n* = 20)
Age (years)		
Mean	43	38
Range	20–75	22–66
Sex		
Male	5	4
Female	6	7
Location	Lesion number (*n*)/ Irradiation mean size (c.c. for HT, cm for Electron)	
Scalp	1/220.5 c.c	
Ear	5/31.1 c.c.	5/5.2 cm
Lip	1/53.2 c.c	
Neck	1/102.1 c.c	1/4.8 cm
Shoulder	4/152.1 c.c	1/6.2 cm
Arm	4/214.5 c.c	2/6.5 cm
Anterior chest wall		2/8.6 cm
Breast		5/9.8 cm
Abdomen	2/700.1 c.c.	1/5.0 cm
Pelvis	4/311.8 c.c.	2/6.0 cm
Back	1/200.2 c.c	
Scapula		1/5.5 cm

Abbreviation: HT: helical tomotherapy.

**Table 2:** Calculated doses to organs at risk and the Paddick conformity index (PCI) and uniformity index (UI) for patients with keloids treated with helical tomotherapy.

Critical Organ	Mean ± SD (Gy)	Maximum ± SD (Gy)
Right lens	-	0.05 ± 0.04
Left lens	-	0.08 ± 0.05
Right eyeball	0.05 ± 0.04	-
Left eyeball	0.07 ± 0.05	-
Right optic nerve	-	0.04 ± 0.03
Left optic nerve	-	0.08 ± 0.06
Optic chiasm	-	0.06 ± 0.01
Right inner ear	0.07 ± 0.06	-
Left inner ear	0.20 ± 0.11	-
Right parotid	0.10± 0.05	-
Left parotid	0.80 ± 0.72	-
Brain	0.26 ± 0.22	-
Heart	1.50 ± 0.32	-
Whole lungs	1.09 ± 0.20	-
Bilateral kidneys	0.55 ± 0.45	-
Liver	1.28 ± 0.23	-
Esophagus	0.80 ± 0.51	-
Trachea	0.78 ± 0.39	-
Intestine	1.75 ± 0.02	-
Stomach	0.85 ± 0.26	-
Bladder	0.35 ± 0.05	-
Rectum	0.19 ± 0.09	-
Uterus	0.28 ± 0.11	-
Bilateral femur bones	0.64 ± 0.43	-
PCI	0.70 ± 0.05	-
UI	1.09 ± 0.3	-

Femur bones included the femoral head and neck. SD, standard deviation; PCI, Paddick conformity index; UI, uniformity index.

**Table 3:** Surface doses delivered to the skin for patients with keloids treated with helical tomotherapy (HT) or electron beam irradiation (13.5 Gy in 3 fractions) as measured with radiochromic EBT3 film.

Treatment Site	HT Group		Electron Beam Group		*p* Value
	Mean Dose (cGy)	Compared to Prescription Dose (%)	Mean Dose (cGy)	Compared to Prescription Dose (%)	
Ear	466.5	+3.7%	417.6	−7.2%	
Lip	502.8	+11.7%	-	-	
Shoulder	483.7	+7.5%	422.6	−6.1%	
Arm	489.3	+8.7%	427.1	−5.1%	
Chest	-	-	429.8	−4.5%	
Abdomen	467.5	+3.9%	430.7	−4.3%	
Pelvis	506.1	+12.5%	438.3	−2.6%	
Mean	486.0	+8.0%	427.5	−5.0%	*p* = 0.001
